# Amino Acid and Mineral Element Analysis and Quality Evaluation of Different Color Fruits of *Nitraria* in Qinghai

**DOI:** 10.1155/ijfo/7405684

**Published:** 2026-04-06

**Authors:** Changmao Chen, Yu Tang, Yang Yang, Xiuhua Guo, Yubi Zhou, Jie Wang

**Affiliations:** ^1^ Qinghai Key Laboratory of Tibetan Medicines Pharmacology and Safety Evaluation, Northwest Institute of Plateau Biology, Chinese Academy of Sciences, Xining, Qinghai, China, cas.cn; ^2^ College of Pharmacy, Qinghai Minzu University, Xining, Qinghai, China, qhmu.edu.cn; ^3^ Forest Seedling Station of Qinghai Province, Forestry and Grassland Administration of Qinghai Province, Xining, Qinghai, China; ^4^ Testing Department, Qinghai PuceTesting Co., Ltd, Xining, Qinghai, China; ^5^ University of Chinese Academy of Sciences, Chinese Academy of Sciences, Beijing, China, cas.cn

**Keywords:** amino acids, mineral elements, *Nitraria roborowskii*, *Nitraria sibirica*, *Nitraria tangutorum*, quality evaluation

## Abstract

Only three species of *Nitraria* fruits are distributed in Qinghai Province, which serve as important berry resources in the desert ecosystem of Qinghai. These fruits are rich in nutrients; however, the differences in nutritional characteristics and quality evaluation among different species and fruit colors have not been systematically clarified. To explore the nutritional quality of *Nitraria* fruits in Qinghai, this study investigated red and purple fruits of *Nitraria roborowskii*, *N. tangutorum*, and *N. sibirica*. The contents of 17 amino acids and 27 mineral elements were determined, analyzed by multivariate chemometric methods, and compared with existing research results for verification. The results showed that the contents of total amino acids, essential amino acids, nonessential amino acids, and total mineral elements in *N. sibirica* were significantly higher than those in *N. roborowskii* and *N. tangutorum*. Among them, the proportion of essential amino acids in purple fruits of *N. sibirica* reached 0.22, close to the FAO ideal protein standard, and it was rich in functional mineral elements such as zinc and iron, showing high nutritional value. These results are consistent with the existing research on the nutritional specialization of desert berries. Fruit color exerted differential effects on nutritional components: nonessential amino acids and total amino acids were positively affected in red fruits, while mineral element contents were not significantly influenced by color, which agrees with studies on metabolic competition related to anthocyanin biosynthesis. Synergistic accumulation was observed between amino acids and mineral elements. Species inheritance was the dominant factor determining nutritional quality. Al, Val, Ca, V, and Cr were screened as core signature components, which improved the indicator system for the quality evaluation of *Nitraria* plants. Comprehensive evaluation demonstrated that *N. sibirica* possessed the best overall nutritional quality, and red fruits were generally superior to purple fruits, with purple fruits of *N. sibirica* exhibiting the optimal nutritional quality. The results of this study provide a theoretical basis for the precise development of *Nitraria* germplasm resources in Qinghai, the breeding of superior genotypes, and the research and development of distinctive functional foods from desert plants.

## 1. Introduction

Plants of the genus *Nitraria* are shrubs widely distributed in extreme environments such as deserts and saline‐alkali lands, and they are key species maintaining the ecological balance in arid regions [1]. Only three *Nitraria* species are distributed in Qinghai Province, including *Nitraria roborowskii* Kom., *N. tangutorum* Bobrov., and *N. sibirica* Pall [2]. Their fruits are rich in nutrients such as amino acids and mineral elements, and fruit color can regulate flavor and nutritional characteristics, thereby affecting fruit quality. These fruits have both edible and medicinal values and exhibit great potential in the development of functional foods and ecological restoration [3–6]. The composition and proportion of amino acids are core indicators for evaluating the nutritional value of food, while mineral elements are involved in the regulation of human physiological functions; the synergistic accumulation of the two is crucial for the functionality of fruits [7–10].

In recent years, with the increasing demand for the development of functional foods, the evaluation of nutritional quality of berries in desert areas has become a research focus. The accumulation of nutritional components in fruits is often jointly affected by species genetic characteristics (species) and phenotypic traits (color) [11–13]. Metabolic differences among species are the core factors for the differentiation of plant nutritional quality. During the long‐term adaptation to extreme environments, different *Nitraria* species may form unique accumulation characteristics of amino acids and mineral elements, which is consistent with the metabolic rules of environmental adaptation of other desert plants [12]. As an intuitive phenotypic marker, fruit color is often associated with pigment synthesis. The biosynthesis of pigments may have precursor competition with amino acid metabolism, which may affect the distribution of nutritional components [14].

At present, existing studies have carried out single‐component analysis of mineral elements and amino acids in *Nitraria* plants [5, 15]. For example, Yuan et al. used inductively coupled plasma (ICP)‐OES to perform principal component analysis (PCA) on mineral elements in *N. roborowskii* fruits from different producing areas, and Gao & Suo determined the amino acid content in *Nitraria* fruits from the Qaidam Basin. However, systematic studies on the differences in amino acid and mineral element compositions, as well as the synergistic accumulation rules, among fruits of different colors from three *Nitraria* species in Qinghai are still lacking. Comprehensive quality evaluation and screening of core signature components based on multivariate chemometrics have not been thoroughly carried out. In addition, most existing studies focus on inland desert areas, and there is still a gap in the research on the nutritional characteristics of *Nitraria* plants in alpine desert areas.

Therefore, this study selected red and purple fruits of three *Nitraria* species (i.e., *N. roborowskii*, *N. tangutorum*, and *N. sibirica*) in Qinghai as research objects. By determining the contents of amino acids and mineral elements, combined with multidimensional chemometric methods, this study aims to reveal the differences in amino acid and mineral element contents among fruits of different *Nitraria* species and colors, clarify the dominant factors affecting the differences in their nutritional quality, screen core signature components, and establish a scientific evaluation system. Meanwhile, the research results were compared and verified with the existing conclusions on the nutrition of desert plants and berries, so as to supplement the nutritional research data of *Nitraria* plants in alpine desert areas and provide a theoretical basis for the precise development of their resources. This study put forward testable hypotheses:① There are significant differences in fruit nutritional components among different *Nitraria* species; *N. sibirica* has higher accumulation of amino acids and mineral elements, which is consistent with the nutritional accumulation rule of superior germplasm of desert plants; ② fruit color has an impact on nutritional components, and the impact rule is consistent with the metabolic competition mechanism of pigment synthesis; and ③ there is a synergistic accumulation effect between amino acids and mineral elements, which is consistent with the research conclusions on the nutritional characteristics of other berries.

## 2. Results and Discussion

### 2.1. Analysis of Amino Acid Content

**FIGURE 1:**
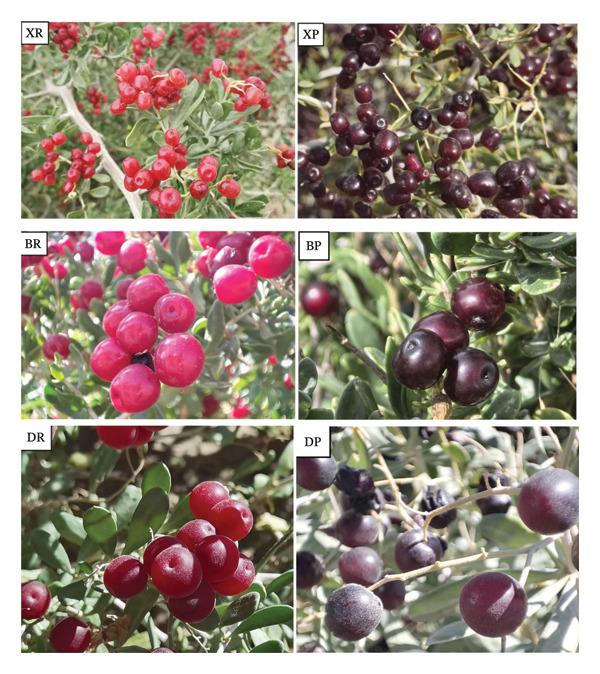
Morphological comparison of *Nitraria* fruits from three species with different fruit colors. Note: DR: *Nitraria roborowskii* red fruit; DP: *Nitraria roborowskii* purple fruit; BR: *Nitraria tangutorum* red fruit; BP: *Nitraria tangutorum* purple fruit; XR: *Nitraria sibirica* red fruit; XP: *Nitraria sibirica* purple fruit.

There are only three species of *Nitraria* in Qinghai, and their red and black (or purple) fruits are shown in Figure 1. In this study, an automatic amino acid analyzer was used to determine 17 amino acids in the dry weight samples of red fruits (R) and purple fruits (P) from three *Nitraria* species, including eight essential amino acids (EAAs) and nine non‐EAAs (NEAAs). The limits of detection (LODs), limits of quantification (LOQ), and corrections for amino acids after hydrolysis are presented in Supporting Tables S2 and S3, respectively. Quality control (QC) information and coefficients of variation (CVs) are shown in Supporting Tables S6 and S7, validating the feasibility of the data. The results showed that species and fruit color had significant effects on amino acid accumulation, which was highly consistent with the conclusions of the existing relevant studies. Details are shown in Table 1.

**TABLE 1:** Contents of nonessential, essential, and total amino acids in *Nitraria* fruits of different colors (mg/kg).

Amino acids	DR	DP	BR	BP	XR	XP
Asp	1160 ± 52.92	400 ± 17.32	1400 ± 75.50	800 ± 34.64	26,300 ± 1212.44	1600 ± 72.11
Ser	900 ± 43.59	400 ± 17.32	1000 ± 43.59	600 ± 26.46	3300 ± 147.31	2000 ± 91.65
Glu	900 ± 43.59	700 ± 36.06	900 ± 43.59	600 ± 26.46	1300 ± 62.45	1500 ± 72.11
Pro	970 ± 45.83	500 ± 26.46	2100 ± 111.36	1500 ± 72.11	13,100 ± 655.74	2900 ± 137.48
Gly	2760 ± 131.15	3800 ± 176.92	6800 ± 327.87	3100 ± 137.48	3200 ± 147.31	7700 ± 347.71
Ala	660 ± 36.06	800 ± 34.64	300 ± 17.32	300 ± 17.32	400 ± 17.32	900 ± 43.59
Cys	9200 ± 437.15	7500 ± 357.63	6800 ± 327.87	1900 ± 81.85	7200 ± 337.79	7900 ± 367.56
Tyr	400 ± 17.32	400 ± 17.32	300 ± 17.32	200 ± 10.00	300 ± 17.32	1000 ± 43.59
Arg	700 ± 36.06	600 ± 26.46	1600 ± 72.11	1100 ± 52.92	1700 ± 81.85	2400 ± 108.17
Nonessential amino acid content	17,650 ± 838.03	15,100 ± 703.78	21,200 ± 1031.16	10,100 ± 456.40	56,800 ± 2676.14	27,900 ± 1279.96
Val	400 ± 17.32	300 ± 17.32	600 ± 26.46	600 ± 26.46	800 ± 34.64	1400 ± 75.50
Met	100 ± 4.36	100 ± 4.36	0 ± 0.00	0 ± 0.00	200 ± 10.00	200 ± 10.00
Ile	200 ± 10.00	100 ± 4.36	200 ± 10.00	300 ± 17.32	300 ± 17.32	500 ± 26.46
Leu	1600 ± 72.11	1300 ± 62.45	1700 ± 81.85	1000 ± 43.59	1800 ± 81.85	2800 ± 127.67
Phe	500 ± 26.46	400 ± 17.32	800 ± 34.64	600 ± 26.46	800 ± 34.64	1300 ± 62.45
His	210 ± 10.00	100 ± 4.36	200 ± 10.00	200 ± 10.00	300 ± 17.32	200 ± 10.00
Lys	300 ± 17.32	300 ± 17.32	500 ± 26.46	500 ± 26.46	700 ± 36.06	700 ± 36.06
Thr	300 ± 17.32	200 ± 10.00	300 ± 17.32	300 ± 17.32	500 ± 26.46	800 ± 34.64
Essential amino acid content	3610 ± 172.57	2800 ± 135.94	4300 ± 204.21	3500 ± 166.43	5400 ± 255.15	7900 ± 380.00
Total amino acid content	21,260 ± 1009.20	17,900 ± 838.14	25,500 ± 1234.06	13,600 ± 622.17	62,200 ± 2926.93	35,800 ± 1659.55

*Note:* The amino acids include nonessential amino acids (Asp, Ser, Glu, Pro, Gly, Ala, Cys, Tyr, Arg) and essential amino acids (Val, Met, Ile, Leu, Phe, His, Lys, Thr). Values are expressed as mean ± standard deviation (*n* = 3). DR: *N. roborowski*i red fruit, DP: *N. roborowskii* purple fruit, BR: *N. tangutorum* red fruit, BP: *N. tangutorum* purple fruit, XR: *N. sibirica* red fruit, XP: *N. sibirica* purple fruit. among the three Nitraria species (*N. roborowskii, N. tangutorum, and N. sibirica*). The amino acid accumulation in Nitraria plants is jointly influenced by species and fruit color. *Nitraria roborowskii* has a significantly superior amino acid accumulation capacity compared with other species. Nonessential amino acids and total amino acids have positive effects on red fruits, while the influence of essential amino acids on fruit color varies with species.

In terms of interspecific differences, the contents of NEAAs, EAAs, and total amino acids (TAA) in red and purple fruits of *N. sibirica* were extremely significantly higher than those in the other two species (Figures 2(a), 2(b), 2(c)) (*p* < 0.01), indicating that *N. sibiric*a had the best amino acid nutritional quality. This is consistent with the conclusion of Liu’s analysis on the nutritional components of *Nitraria* plants in Inner Mongolia that *N. sibirica* is a species with superior nutritional accumulation capacity in the genus *Nitraria* and also conforms to the general rule that desert plants enhance their stress resistance through the accumulation of high levels of metabolic substances. *N. sibirica* enhances the stress resistance of seed germination by accumulating a large amount of amino acids, which is consistent with the research results that exogenous proline alleviates low‐temperature stress in maize and improves the stress resistance of desert plants[16, 17]. In addition, the ratios of EAAs to TAA in red and purple fruits of *N. sibirica* were 8.6% and 22%, respectively. The proportion of EAAs in purple fruits of *N. sibirica* was higher and close to the FAO ideal protein standard. This result supplements the research on the amino acid content of *Nitraria* in the Qaidam Basin by Gao & Suo et al., adds data on the amino acid proportion characteristics of *N. sibirica* fruits of different colors in alpine regions, and confirms that *N. sibirica* has more advantages in amino acid nutritional value [15, 16].

FIGURE 2Comparative analysis of nonessential, essential, and total amino acid contents in *Nitraria* fruits grouped by species and fruit color. Note: (a) nonessential amino acid contents grouped by species; (b) essential amino acid contents grouped by species; (c) total amino acid contents grouped by species; (d) nonessential amino acid contents grouped by fruit color; (e) essential amino acid contents grouped by fruit color; (f) total amino acid contents grouped by fruit color. Values are expressed as mean ± standard deviation (*n* = 3). ^∗∗^indicates significant difference at *p* < 0.01 level; ^∗^indicates significant difference at *p* < 0.05 level. DR: *N. roborowskii* red fruit, DP: *N. roborowskii* purple fruit, BR: *N. tangutorum* red fruit, BP: *N. tangutorum* purple fruit, XR: *N. sibirica* red fruit, XP: *N. sibirica* purple fruit. D: *N. roborowskii* fruit, B: *N. tangutorum* fruit, X: *N. sibirica* fruit. When grouped by species, the red fruits (DR, BR, XR) consistently contain significantly higher levels of nonessential, essential, and total amino acids than the purple fruits (DP, BP, XP) of the same species, as indicated by the double asterisks (^∗∗^) denoting significant differences at *p* < 0.01. When grouped by fruit color, this pattern is further confirmed: across all three *Nitraria* species, red fruits (R) show significantly higher nonessential, essential, and total amino acid contents than purple fruits (P). This highlights that fruit color is a key factor associated with amino acid accumulation in *Nitraria* fruits, with red fruits generally exhibiting superior nutritional value in terms of amino acid content.

**Figure:**
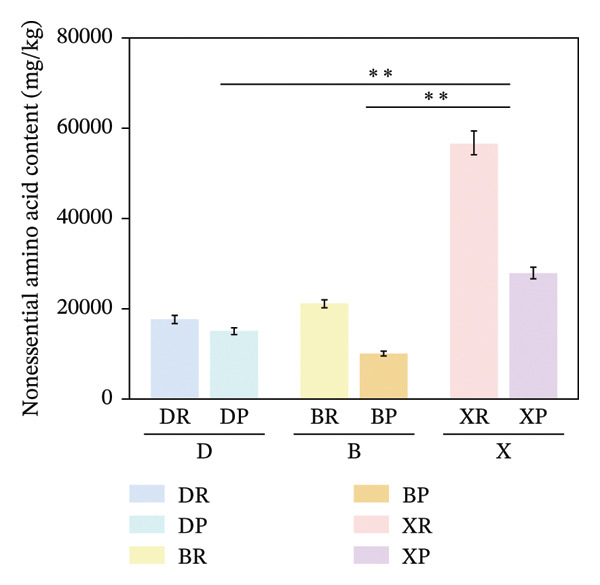
(a)

**Figure:**
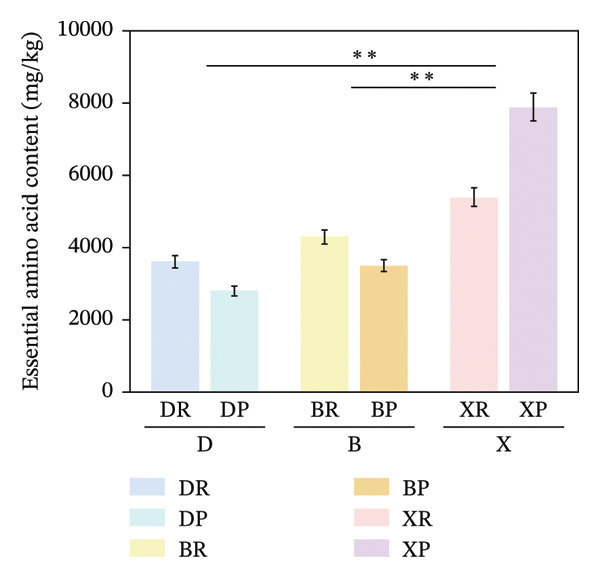
(b)

**Figure:**
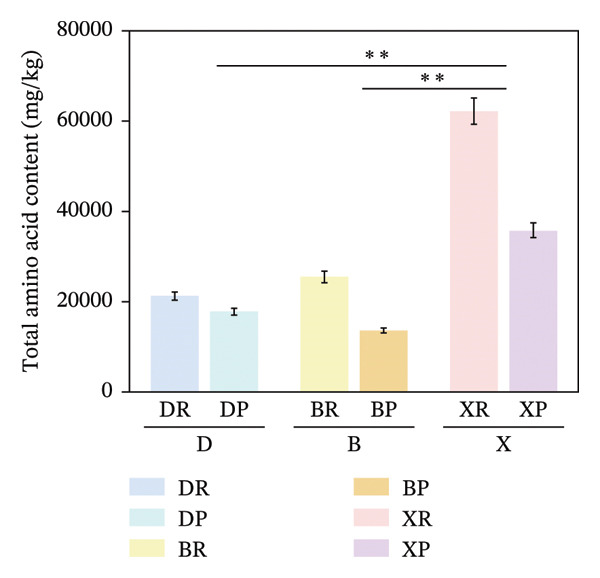
(c)

**Figure:**
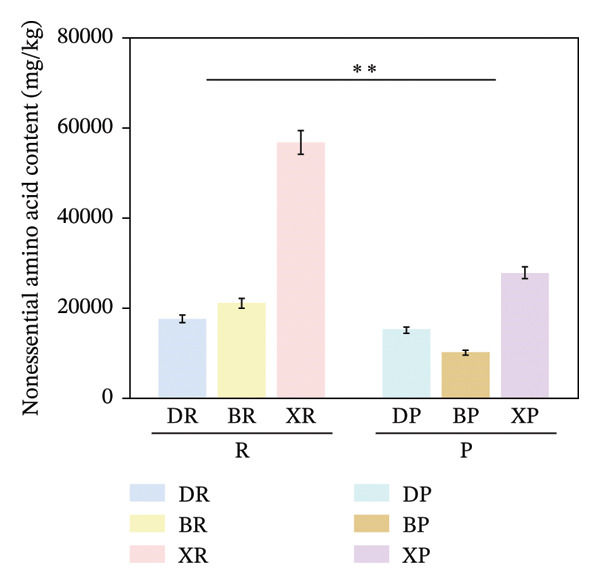
(d)

**Figure:**
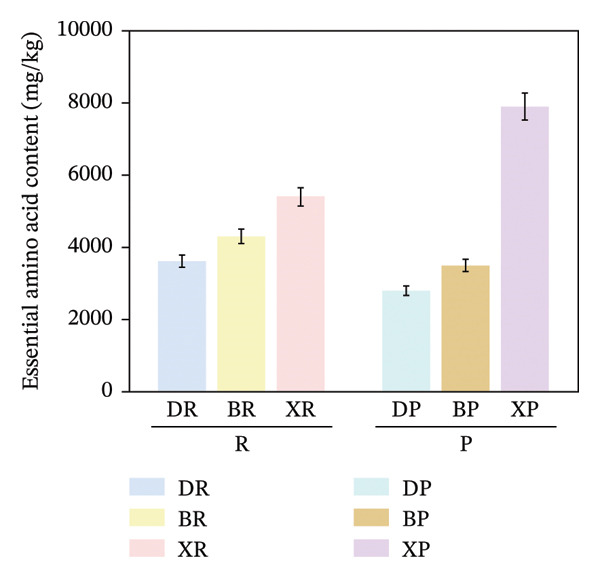
(e)

**Figure:**
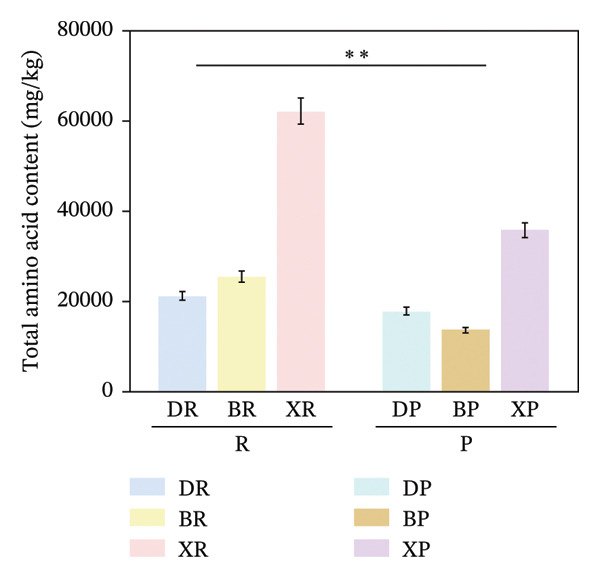
(f)

In terms of color differences, the contents of NEAAs and TAA in red fruits were significantly higher than those in purple fruits (*p* < 0.01) (Figure 2). Except for *N. sibirica*, the EAA contents in red fruits of *N. roborowskii* and *N. tangutorum* were higher than those in their respective purple fruits (Figure 2). These results indicate that red fruits have better amino acid nutritional quality. The color difference in EAAs varied with species, which is consistent with the research conclusions related to metabolic competition in anthocyanin synthesis [18, 19]. The purple color of purple fruits is derived from anthocyanins, and their synthesis requires the consumption of amino acids such as phenylalanine as precursors, leading these amino acids to flow more into the pigment synthesis pathway rather than accumulating in free form. This mechanism has been confirmed in studies on the relationship between anthocyanins and nutritional components in purple corn kernels and eucalyptus [18, 19]. However, EAAs did not show a consistent color difference pattern, indicating that their accumulation is more strongly regulated by the metabolic characteristics of the species themselves, which is consistent with the conclusion proposed by Yu et al. that the effect of fruit color on nutritional components is species‐specific [18].

In summary, the amino acid accumulation of *Nitraria* plants is jointly affected by species and fruit color. *N. sibirica* has significantly superior amino acid accumulation capacity compared with other species; NEAAs and TAA have positive effects on red fruits, while the effect of EAAs on fruit color varies with species. These results not only verify the existing characteristics of amino acid differences in *Nitraria* fruits of different colors in alpine desert areas but also provide data support for the systematic study of amino acid nutrition in *Nitraria* plants.

### 2.2. Analysis of Mineral Element Composition

Mineral elements are crucial for plant growth and development, and ensuring appropriate nutrient supply is the key to achieving an optimal fruit yield and quality [20]. In this study, ICP‐mass spectrometry (ICP‐MS) and ion chromatography were employed to determine the mineral elements in the dry weight samples of red fruits (R) and purple fruits (P) from three *Nitraria* species. Information on the internal standards and method validation parameters during the analytical procedure is presented in supporting Tables S4 and S5, and the CV values are shown in supporting Table S8, confirming the reliability and feasibility of the data. The results showed that *N. sibirica* was the dominant species in mineral element accumulation, and fruit color had no significant effect on mineral elements, which is consistent with the existing research rules on mineral elements in desert plants [21]. Details are shown in Table 2.

**TABLE 2:** Contents of macromineral and micromineral elements in *Nitraria* fruits of different colors (mg/kg).

Element	DR	DP	BR	BP	XR	XP
Ca	227.67 ± 9.61	129.33 ± 0.58	117.67 ± 6.03	124.33 ± 0.58	172 ± 3.46	164.33 ± 2.31
K	2159 ± 42.51	1832.33 ± 15.95	2491 ± 22.91	1879.33 ± 39.58	2589.33 ± 267.28	3393.3333 ± 80.75
P	179 ± 5.00	146.33 ± 1.15	289 ± 7.81	117.33 ± 0.58	252.67 ± 3.79	280 ± 2.65
S	142.67 ± 4.04	99.87 ± 0.23	160.67 ± 5.77	186.67 ± 1.53	129 ± 1.73	221.33 ± 4.93
Mg	177 ± 1.73	131.33 ± 0.58	208 ± 66.84	279.33 ± 2.08	284.33 ± 4.04	296 ± 3.61
Na	2730 ± 36.06	2740 ± 30.00	2323.33 ± 127.41	3120 ± 20.00	3836.67 ± 25.17	3410 ± 50.00
Cl^-^	271 ± 2.00	266 ± 2.00	520 ± 20.00	217 ± 2.00	603 ± 13.00	425 ± 3.00
Macronutrient content	5886.33 ± 52.27	5345.2 ± 45.18	6109.67 ± 122.32	5924 ± 56.79	7867 ± 274.25	8190 ± 86.03
V	0.01 ± 10.50*E* − 4	0.01 ± 8.50*E* − 4	0.02 ± 10.60*E* − 4	0.01 ± 0.30*E* − 4	0.02 ± 10.20*E* − 4	0.03 ± 14.60*E* − 4
Cr	0.03 ± 4.90*E* − 4	—	—	0.02 ± 24.80*E* − 4	—	0.02 ± 0.01
Co	0.01 ± 1.00*E* − 4	0.01 ± 1.50*E* − 4	0.02 ± 2.30*E* − 4	0.02 ± 0.40*E* − 4	0.04 ± 1.20*E* − 4	0.07 ± 4.70*E* − 4
Cu	0.7 ± 0.04	0.6 ± 0.03	0.48 ± 0.01	0.5 ± 0.01	0.65 ± 45.80*E* − 4	0.69 ± 0.01
Fe	7.02 ± 0.18	8.6 ± 0.39	4.99 ± 0.06	5.09 ± 0.09	7.3 ± 0.13	11.23 ± 0.15
Mn	1.38 ± 0.02	1.32 ± 0.05	1.38 ± 0.02	1.8 ± 0.04	2.51 ± 57.70*E* − 4	2.38 ± 0.04
F^-^	7.49 ± 0.14	4.74 ± 0.09	7.62 ± 0.12	10 ± 0.1	11 ± 0.1	6.46 ± 0.11
Ti	0.19 ± 15.30*E* − 4	0.13 ± 10.00*E* − 4	0.1 ± 18.00*E* − 4	0.07 ± 8.30*E* − 4	0.17 ± 10.00*E* − 4	0.19 ± 15.30*E* − 4
Sr	3.18 ± 0.03	1.21 ± 0.02	1.19 ± 0.02	1.62 ± 0.03	3.02 ± 0.01	2.12 ± 0.02
Li	0.08 ± 2.90*E* − 4	0.09 ± 14.40*E* − 4	0.1 ± 31.10*E* − 4	0.26 ± 43.60*E* − 4	0.09 ± 2.10*E* − 4	0.11 ± 0.00
B	1.27 ± 0.01	0.84 ± 0.01	0.92 ± 0.03	0.9 ± 0.02	1.07 ± 0.02	1.56 ± 0.01
Al	9.52 ± 0.77	9.04 ± 0.5	2.15 ± 0.17	3.44 ± 0.12	4.53 ± 0.04	6.92 ± 0.24
Nd	0.08 ± 8.00*E* − 4	0.03 ± 9.50*E* − 4	0.02 ± 4.60*E* − 4	0.02 ± 0.40*E* − 4	0.05 ± 3.50*E* − 4	0.05 ± 1.70*E* − 4
Y	0.05 ± 7.80*E* − 4	0.02 ± 2.60*E* − 4	0.01 ± 0.60*E* − 4	0.01 ± 1.20*E* − 4	0.04 ± 5.30*E* − 4	0.03 ± 8.10*E* − 4
La	0.10 ± 6.70*E* − 4	0.04 ± 8.30*E* − 4	0.02 ± 4.20*E* − 4	0.02 ± 1.50*E* − 4	0.06 ± 9.00*E* − 4	0.06 ± 9.70*E* − 4
Ce	0.18 ± 15.30*E* − 4	0.07 ± 11.70*E* − 4	0.04 ± 17.10*E* − 4	0.03 ± 9.80*E* − 4	0.11 ± 20.00*E* − 4	0.12 ± 0.00
NO_3_^-^	0.64 ± 0.01	0.49 ± 0.01	0.54 ± 0.01	0.48 ± 0.01	0.67 ± 120.00*E* − 4	0.49 ± 0.01
SO_4_^2-^	27.50 ± 0.30	13.3 ± 0.20	14.6 ± 0.20	17.4 ± 0.20	20.7 ± 0.20	28.9 ± 0.20
PO_4_^3-^	6.03 ± 0.13	7.37 ± 0.17	5.48 ± 0.10	5.87 ± 0.10	5.67 ± 0.10	5.69 ± 0.10
Cd	0.01 ± 1.60*E* − 4	0.01 ± 1.00*E* − 4	0 ± 0.80*E* − 4	0 ± 0.70*E* − 4	0.01 ± 1.50*E* − 4	0.02 ± 7.50*E* − 4
Micronutrient content	65.48 ± 1.33	47.92 ± 0.52	39.67 ± 0.24	47.56 ± 0.27	57.69 ± 0.29	67.17 ± 0.08
Total element content	5951.81 ± 51.25	5393.12 ± 45.51	6149.34 ± 122.55	5971.56 ± 57.06	7924.69 ± 274.14	8257.17 ± 85.96

*Note:* The table includes macronutrients (Ca, K, P, S, Mg, Na, Cl) and micronutrients (V, Cr, Co, Cu, Fe, Mn, F^−^,Ti, Sr, Li, B, Al, Nd, Y, La, Ce, NO_3_^−^, SO_4_^2−^, PO_4_^3−^,Cd). Values are expressed as mean ± standard deviation (*n* = 3). DR: *N. roborowski*i red fruit, DP: *N. roborowskii* purple fruit, BR: *N. tangutorum* red fruit, BP: *N. tangutorum* purple fruit, XR: *N. sibirica* red fruit, XP: *N. sibirica* purple fruit. Among the three *Nitraria* species (*N. roborowskii, N. tangutorum, and N. sibirica*), red fruits (DR, BR, XR) consistently contain higher contents of macronutrients, micronutrients, and total mineral elements than purple fruits (DP, BP, XP) of the same species. This trend is consistent across all tested samples, further confirming that red fruits exhibit superior nutritional quality in terms of mineral composition.

In terms of interspecific differences, the contents of macroelements, microelements, and total mineral elements in red and purple fruits of *N. sibirica* were significantly higher than those in *N. roborowskii* and *N. tangutorum* (Figure 3) (*p* < 0.01, *p* < 0.05). This indicates that *N. sibirica* has the best nutritional quality in terms of mineral elements, which is consistent with the conclusion proposed by Hou et al. that *N. sibirica* has strong adaptability to salt‐alkali and heavy metal stress and possesses a unique physiological mechanism for mineral element accumulation, confirming that the high accumulation capacity of mineral elements is an inherent metabolic characteristic of *N. sibirica* [22]. *N. sibirica* is enriched in functional microelements such as Zn and Fe, which is consistent with the research conclusion put forward by Ma et al. that Zn and Fe are core functional mineral elements of berries and important indicators for the development of iron and zinc supplement functional foods, indicating that *N. sibirica* has greater potential in the development of nutritional and functional foods [23]. Meanwhile, its characteristic of high K and Ca contents in macroelements not only conforms to the physiological mechanism of desert plants proposed by Joshi et al. that K regulates osmotic pressure and Ca stabilizes cell walls to enhance plant stress resistance but also improves the edible value of fruits due to the physiological functions of Ca in preventing osteoporosis and K in assisting blood pressure control. This result supplements the research on mineral elements only focusing on *N. roborowskii* by Yuan et al. and improves the characteristics of mineral element accumulation in three *Nitraria* species in Qinghai Province [5, 24].

FIGURE 3Comparative analysis of macromineral, micromineral, and total element contents in *Nitraria* fruits grouped by species and fruit color. Note: (a) macronutrient contents grouped by species; (b) micronutrient contents grouped by species; (c) total element contents grouped by species; (d) macronutrient contents grouped by fruit color; (e) micronutrient contents grouped by fruit color; (f) total element contents grouped by fruit color. Values are expressed as mean ± standard deviation (*n* = 3). ^∗∗^indicates significant difference at *p* < 0.01 level; ^∗^indicates significant difference at *p* < 0.05 level. DR:*N. roborowskii* red fruit, DP: *N. roborowskii* purple fruit, BR: *N. tangutorum* red fruit, BP: *N. tangutorum* purple fruit, XR: *N. sibirica* red fruit, XP: *N. sibirica* purple fruit. D: *N. roborowskii* fruit, B: *N. tangutorum* fruit, X: *N. sibirica* fruit. Among the three *Nitraria* species (*N. roborowskii*, *N. tangutorum*, and *N. sibirica*), the contents of macronutrients, micronutrients, and total mineral elements in red fruits (DR, BR, XR) are consistently and significantly higher than those in purple fruits (DP, BP, XP) of the same species, further confirming that red fruits exhibit superior nutritional quality in terms of mineral composition.

**Figure:**
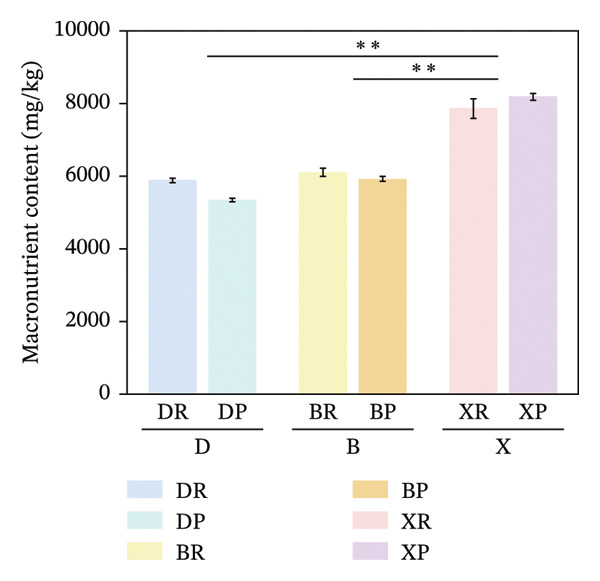
(a)

**Figure:**
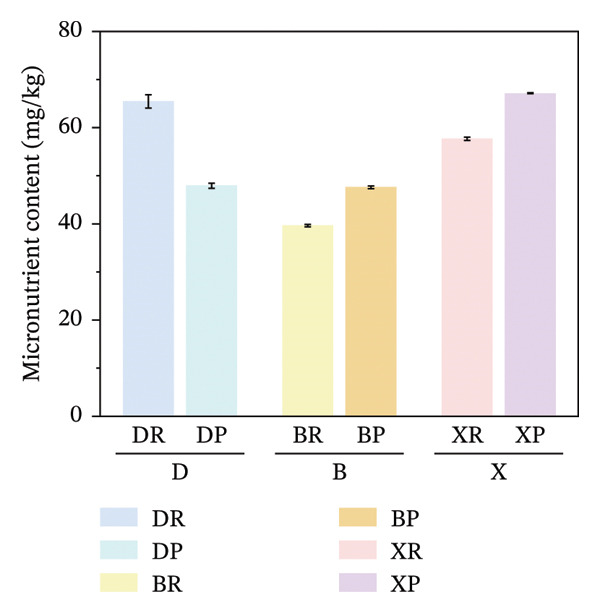
(b)

**Figure:**
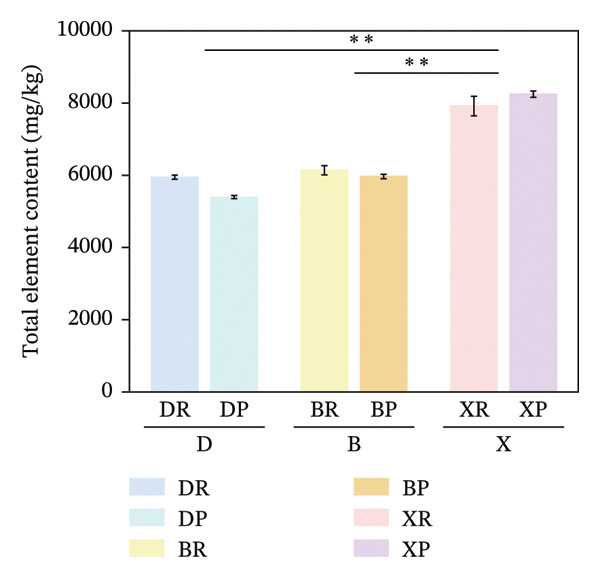
(c)

**Figure:**
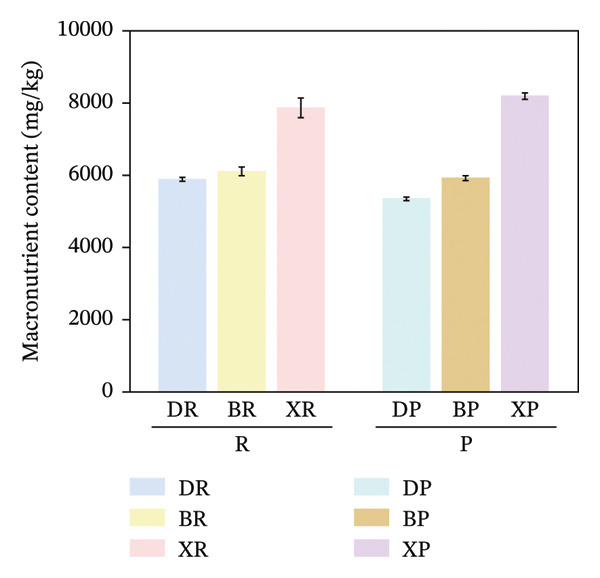
(d)

**Figure:**
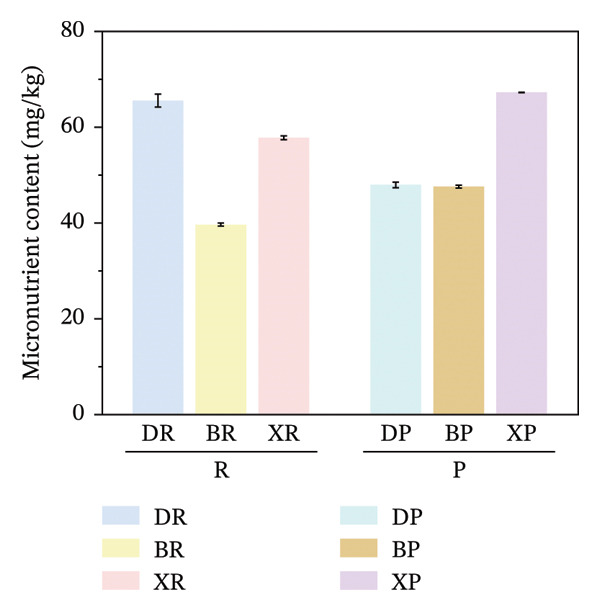
(e)

**Figure:**
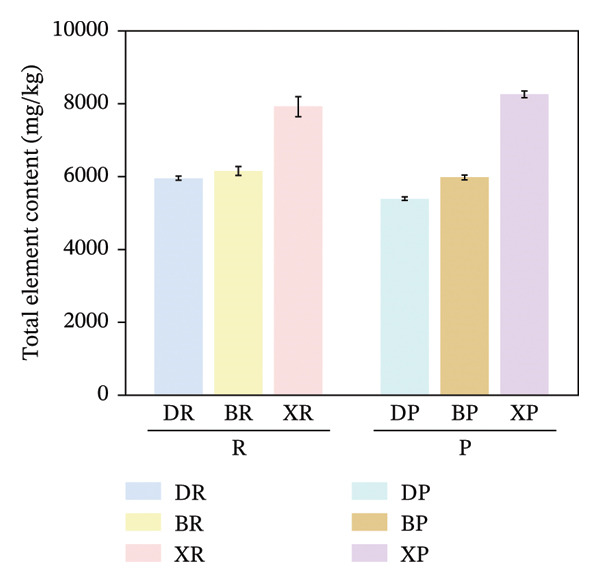
(f)

In terms of color differences, the mineral element contents of fruits of different colors among the three *Nitraria* species did not show statistically significant differences. The mineral element content of purple fruits of *N. sibirica* was slightly higher than that of red fruits, and that of red fruits of *N. roborowskii* was slightly higher than that of purple fruits. This trend was consistent in all tested samples, further confirming that red fruits exhibit better nutritional quality in terms of mineral composition. This result is consistent with the research conclusion of Zhao et al. on the relationship between plum peel color and mineral elements, confirming that mineral elements are not the key factor affecting fruit color. Unlike amino acids, which are significantly affected by color, the content of mineral elements is more dominated by the characteristics of the species itself [25]. This conclusion makes up for the deficiency in the existing research on *Nitraria* regarding the relationship between color and mineral elements and clarifies that the core influencing factor of mineral element accumulation in *Nitraria* plants is species heredity.

### 2.3. Quality Evaluation of Fruits of Different Colors in *Nitraria* Plants by Chemometric Methods

#### 2.3.1. Correlation Analysis

**FIGURE 4:**
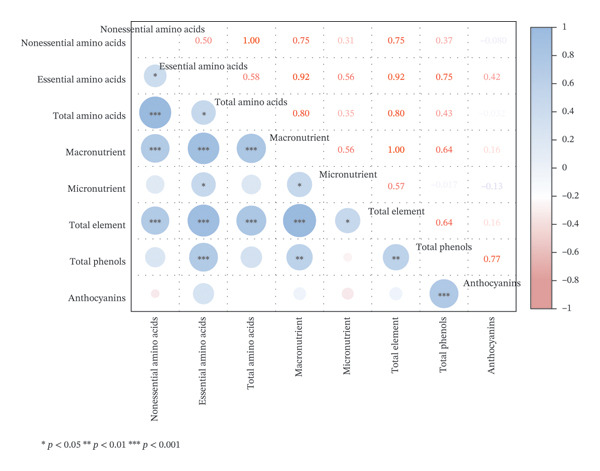
Heatmap of correlation analysis between nutritional components in *Nitraria* fruits of different colors. Note: the heatmap shows the Pearson correlation coefficients between nonessential amino acids, essential amino acids, total amino acids, macromineral elements, micromineral elements, total mineral elements total phenols, and anthocyanins. The color gradient represents the strength and direction of correlations (blue: positive; red: negative). ^∗^indicates the significant correlation at *p* < 0.05 level; ^∗∗^indicates the significant correlation at *p* < 0.01 level; ^∗∗∗^indicates the significant correlation at *p* < 0.001 level. From the heatmap, consistent correlation features with the aforementioned patterns can be observed: Significant positive correlations (*p* < 0.001) exist between amino acid components (nonessential, essential, and total amino acids) and mineral elements (macronutrients, micronutrients, and total mineral elements), indicating that the accumulation of amino acids and mineral nutrients in the fruits is synergistic. Total phenols and anthocyanins also show significant positive correlations with amino acids and mineral elements. The strong positive correlation between anthocyanins and both total amino acids and total mineral elements (*r* = 0.77, *p* < 0.001) further explains the intrinsic link that red fruits (with higher anthocyanin content) exhibit superior amino acid and mineral nutritional quality.

Correlation analysis showed that there were significant positive correlations among six types of nutritional components in *Nitraria* fruits, including EAAs, non‐EAAs (NEAAs), macroelements, microelements, TAAs, and total mineral elements (*p* < 0.05) (Figure 4). Among them, total mineral elements and macroelements were extremely significantly positively correlated with all types of amino acids (*p* < 0.001), and EAAs were significantly positively correlated with microelements (*r* = 0.56, *p* < 0.05), confirming the synergistic accumulation effect between amino acids and mineral elements. This result is highly consistent with the conclusion proposed by Goff that amino acids can promote the absorption of mineral elements and there is a synergistic accumulation rule between them in plants and also consistent with the research results of Ahlstrand et al. on Zn–amino acid complexes. It confirms the holistic characteristics of amino acids and mineral elements in berry nutritional components, supplements the synergistic accumulation rule of these two core nutritional components in *Nitraria* plants, and improves the nutritional synergy research system of desert berries [26, 27]. At the same time, both anthocyanin and total polyphenol contents affect fruit nutritional components, and the determination results are shown in Supporting Table 1. Total phenols and anthocyanins were also extremely significantly positively correlated with all types of amino acids and mineral elements, indicating that the accumulation of nutritional components and active components in *Nitraria* fruits is synergistic, which is consistent with the research conclusion proposed by Bonin et al. that nutritional components and active components show a synergistic change characteristic during fruit ripening [11]. This indicates that the above indicators have a highly consistent change trend in *Nitraria* fruits, and the contents of various nutritional and active components show a synergistic change characteristic, further explaining the internal connection that red fruits (with higher anthocyanin content) have better amino acid and mineral nutritional quality.

#### 2.3.2. Hierarchical Cluster Analysis

**FIGURE 5:**
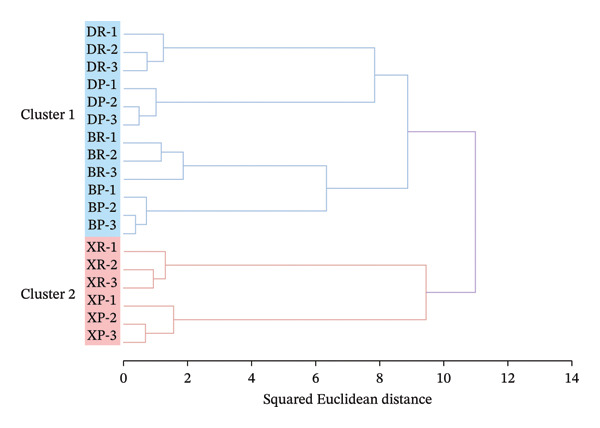
Hierarchical cluster analysis of nutritional components in *Nitraria* fruits of different colors. Note: the dendrogram is based on the squared Euclidean distance, showing that samples are divided into two main clusters: Cluster 1 includes *N. roborowskii* and *N. tangutorum* fruits, while Cluster 2 includes *N. sibirica* fruits. DR: *N. roborowski*i red fruit, DP: *N. roborowskii* purple fruit, BR: *N. tangutorum* red fruit, BP: *N. tangutorum* purple fruit, XR: *N. sibirica* red fruit, XP: *N. sibirica* purple fruit. From the hierarchical cluster analysis, a clear clustering pattern consistent with species classification can be observed: All samples are divided into two main clusters based on the squared Euclidean distance. Cluster 1 consists of *N. roborowskii* and *N. tangutorum* fruits, while Cluster 2 contains only *N. sibirica* fruits. Within each cluster, red and purple fruits of the same species are grouped closely together, indicating that the nutritional component profiles are more similar within the same species, and the difference between fruit colors is secondary to the interspecies difference.

Hierarchical cluster analysis (Figure 5) performed using between‐group average linkage and squared Euclidean distance (SED) showed that when SED was 11, the samples were clustered into two groups: Cluster 1 included red and purple fruits of *N. roborowskii* and *N. tangutorum,* while Cluster 2 included red and purple fruits of *N. sibirica*. No significant differences were observed between samples of different colors within the same species, indicating that the effect of fruit color on the contents of amino acids and mineral elements was less than that of species heredity, and the latter was the key factor affecting the accumulation pattern of these core nutrients. This result is consistent with the conclusion proposed by Saradadevi et al. that species heredity is the core factor for the differentiation of plant nutritional quality, followed by the influence of phenotypic traits, and also aligns with the research rules on crop quality differences reported by Deng et al. [12, 13]. Meanwhile, the clustering results indicated that amino acid and mineral element data could assist in species identification, which provides a new basis for the differentiation of *Nitraria* germplasm resources and makes up for the deficiency that existing identification of *Nitrar*ia germplasm resources only relies on morphological characteristics [28]. In addition, red and purple fruits of *N. sibirica* in Cluster 2 exhibited stronger synergistic accumulation characteristics of nutrients, confirming that they were the group with the optimal nutritional quality. This is consistent with the analysis results of interspecific differences mentioned earlier and conforms to the nutritional characteristic rules of superior germplasm of desert plants [17, 22], further verifying that red fruits have better nutritional quality.

#### 2.3.3. PCA

PCA based on the contents of 17 amino acids and 27 mineral elements showed (Figure 6) that the first three principal components cumulatively explained 85.30% of the total variance, and the samples of the three *Nitraria* species exhibited obvious species separation in the principal component space. This result is consistent with the application conclusions of PCA in the quality evaluation of berries such as jujube and sea buckthorn, confirming that PCA can effectively achieve a dimensionality reduction of nutritional data and species differentiation of *Nitraria* fruits [10, 29].

FIGURE 6Principal component analysis (PCA) of nutrient components in *Nitraria* fruits of different colors. Note: (a) 3D score plot showing the distribution of samples and nutrient components along the first three principal components (PC1: 45.96%, PC2: 23.04%, PC3: 16.31%). (b) 2D biplot showing the contribution of individual nutrients to PC1 and PC2, and the clustering of samples by fruit color and species. DR: *N. roborowski*i red fruit, DP: *N. roborowskii* purple fruit, BR: *N. tangutorum* red fruit, BP:*N. tangutorum* purple fruit, XR: *N. sibirica* red fruit, XP: *N. sibirica* purple fruit. From the principal component analysis (PCA), consistent clustering features with the aforementioned patterns can be observed: samples are clearly separated by species, with *N. sibirica* fruits (XR, XP) forming an independent cluster, which is distinctly separated from *N. roborowskii* (DR, DP) and *N. tangutorum* (BR, BP) fruits, corroborating the results of the hierarchical cluster analysis. Within each species, red fruits (DR, BR, XR) are clearly separated from purple fruits (DP, BP, XP) along the first principal component (PC1). Red fruits show higher positive loadings on nutritional indicators such as amino acids and mineral elements, further confirming that red fruits exhibit superior nutritional quality.

**Figure:**
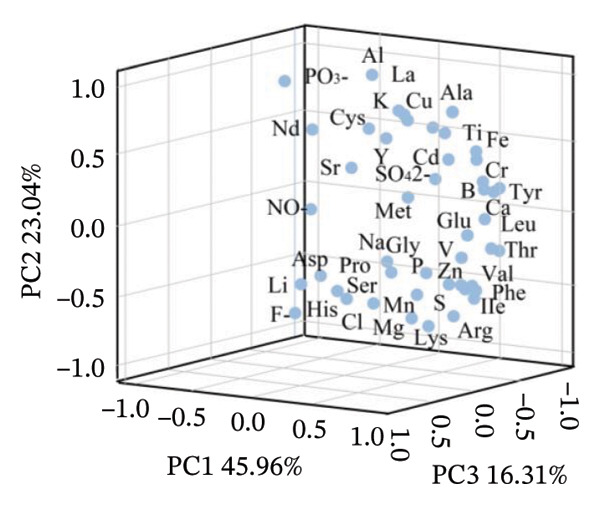
(a)

**Figure:**
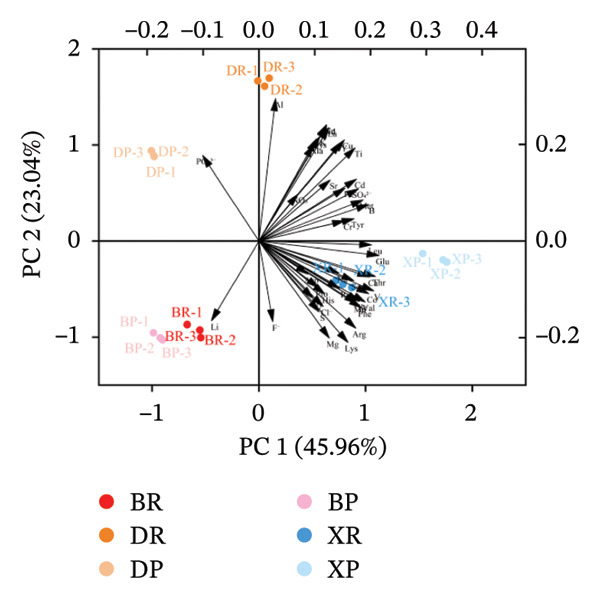
(b)

Samples of red and purple fruits of *N. sibirica* were independently clustered in the positive axis region of PC1, with the core associated indicators being Ca, Mg, Ser, and Pro. The stress resistance + nutrition synergy mechanism reflected by the Ca‐Ser combination was similar to the integrated stress resistance and nutrition metabolic characteristics of sea buckthorn proposed by Wang et al. The physiological functions of Ca in stabilizing the cell wall structure and Ser in enhancing antioxidant capacity not only endow fruits with storage tolerance but also improve their nutritional value. This mechanism improves the research on the synergy between stress resistance and nutrition of berries in alpine desert areas [29, 30]. Samples of *N. roborowskii* were clustered in the positive axis region of PC2, showing a strong correlation with indicators such as Al, PO_3_^–^, Zn, and Ala. The nutritional adaptation strategy reflected by the Al‐PO_3_^–^–Zn combination is consistent with the research conclusion proposed by Deng et al. that plants adapt to saline‐alkali environments by improving phosphorus availability and relying on Zn to maintain metabolic enzyme activity [31]. Samples of *N. tangutorum* were concentrated in the negative axis regions of PC1 and PC2, closely related to indicators such as Li, F^−^, and Lys. The survival strategy for extreme arid environments corresponding to the F^−^–Lys combination not only verifies the physiological mechanism proposed by Meng & Wu that fluoride stabilizes the plant membrane structure to reduce nutrient loss but also aligns with the research results of Sun Yi on lysine ensuring protein synthesis, indicating that *N. tangutorum* can maintain basic nutrient supply in extreme aridity through this mechanism [32, 33].

The clustering results of PCA were completely consistent with those of hierarchical cluster analysis, further confirming that amino acids and mineral elements are the core indicators for distinguishing the fruit quality of different species in the genus *Nitraria.* Meanwhile, it revealed that the differences in nutritional metabolism among the three *Nitraria* species are the evolutionary results of long‐term adaptation to habitats, which is consistent with the conclusion proposed by Saradadevi et al. that plant nutritional specialization is driven by habitats. This provides a nutritional basis for the study on the habitat adaptability of *Nitraria* plants in alpine desert areas [13]. Within the same species, red fruits (DR, BR, XR) and purple fruits (DP, BP, XP) were clearly separated along the direction of the first principal component (PC1), and red fruits had higher positive correlation loads with nutritional indicators such as amino acids and mineral elements, further confirming that red fruits have better nutritional quality.

#### 2.3.4. Neural Network Analysis

**FIGURE 7:**
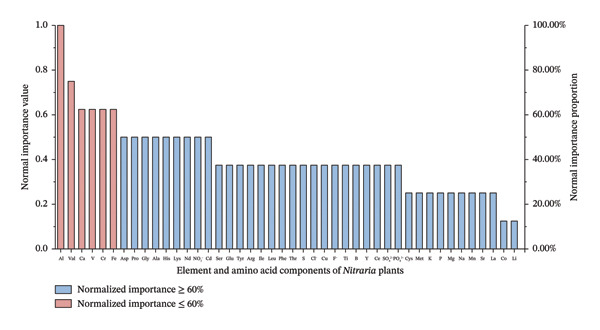
Neural network analysis of nutritional component importance in *Nitraria* fruits of different colors. Note: the bar plot displays the normalized importance values of mineral elements and amino acids. Components are colored by their normalized importance: red bars represent the nutrients with normalized importance ≥ 60%, while blue bars represent those with normalized importance ≤ 60%. This analysis identifies the key nutritional components contributing to the differentiation of *Nitraria* fruits by color. From the neural network importance analysis, key component features consistent with the aforementioned patterns can be observed: Components such as aluminum (Al), valine (Val), cysteine (Cys), vanadium (V), and chromium (Cr) exhibit normalized importance values exceeding 60%, serving as the core nutritional indicators that drive the differentiation of Nitraria fruits by color. These key components are mostly amino acids and mineral elements, further confirming that the synergistic accumulation of amino acids and mineral nutrients is the core driver behind the superior nutritional quality observed in red fruits.

Signature components are the core indicators for food quality evaluation [34]. In this study, as shown in Figure 7, neural network analysis was used to screen Al, Val, Ca, V, and Cr as the core signature components for evaluating the fruit quality of *Nitraria* plants. The neural network model divided the training set and validation set at a ratio of 3:1, and the classification accuracy of both the training set and validation set reached 83.3%, which not only effectively verified the reliability of the preset quality classification (Cluster 2: XR, XP; Cluster 1: DR, DP, BR, BP) but also highlighted the strong discriminant ability of the model.

Among them, Val, as an EAA for humans, is a core raw material for protein synthesis and muscle repair, which is consistent with the research results of Winn et al. on the physiological functions of EAAs [35]. Ca, as an essential macronutrient for humans, is the basis for maintaining bone health and normal cardiovascular function, which aligns with the research conclusion of Giedyk and Gryko [36]. Trivalent Cr can regulate blood glucose levels by enhancing insulin activity, which is consistent with the research results of Jiang et al. on the physiological functions of Cr. These three components together constitute the core basis of the nutritional value of *Nitraria* fruits, which is consistent with the existing screening rules of signature nutritional components in berries [10, 34, 37]. The high importance of Al and V is more reflected as metabolic signature characteristics of *Nitraria* plants adapting to saline‐alkali habitats, which is consistent with the conclusion mentioned by Deng et al. that Al is a metabolic marker for plants’ adaptation to saline‐alkali environments, supplementing the research on signature components for the habitat adaptability of desert plants [31, 38].

In addition, this study found that Cr is a key toxic risk factor that needs to be focused on in the quality evaluation of *Nitraria* fruits. Its multidimensional negative effects on fruit quality are consistent with the research conclusion reported by Rolić et al. that heavy metal elements interfere with plant mineral element and amino acid metabolism and reduce food safety [39]. However, the cadmium (Cd) content in all samples did not exceed the national food safety standard of China (GB2762‐2022). This detection result provides basic data for the food safety risk assessment of *Nitraria* fruits and meets the requirement of conducting systematic heavy metal risk assessment for the development of characteristic berries [40]. The five core signature components screened in this study improve the screening system for signature components of health food proposed by Wang et al. and make up for the lack of core indicators in existing quality evaluation of *Nitraria* plants, providing a scientific basis for the quality grading of *Nitraria* germplasm resources and the breeding of superior varieties [5, 15, 34]. Most of these key components are amino acids and mineral elements, further confirming that the synergistic accumulation of amino acids and mineral nutrients is the core driving factor for red fruits to exhibit better nutritional quality.

#### 2.3.5. Membership Function Method

**FIGURE 8:**
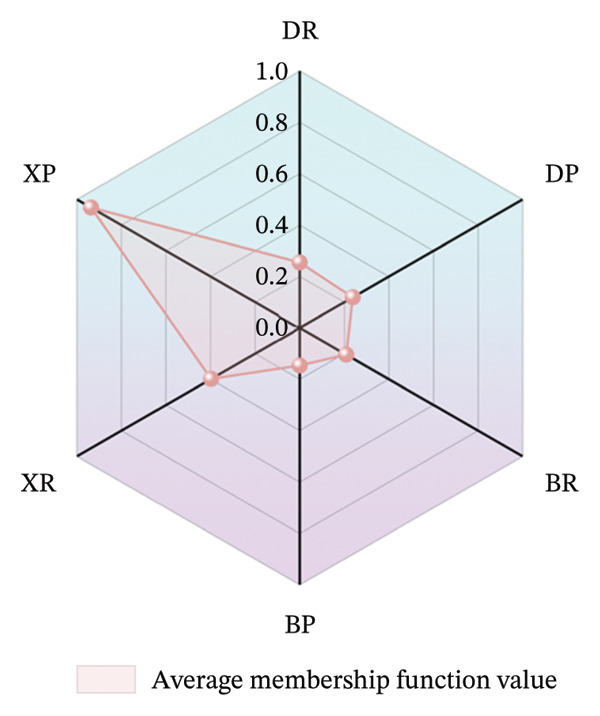
Average membership index values of nutritional components in *Nitraria* fruits of different colors. Note: the radar chart displays the average membership function values of nutritional components across six *Nitraria* fruit groups, reflecting the comprehensive nutritional quality of each group. DR: *N. roborowski*i red fruit, DP: *N. roborowskii* purple fruit, BR: *N. tangutorum* red fruit, BP: *N. tangutorum* purple fruit, XR: *N. sibirica* red fruit, XP: *N. sibirica* purple fruit. From the average membership analysis in the radar chart, comprehensive nutritional quality features consistent with the aforementioned patterns can be observed: The average membership values of *N. sibirica* red fruits (XR) and purple fruits (XP) are significantly higher than those of other groups, indicating that their comprehensive nutritional quality is the best. Within each species, the average membership values of red fruits (DR, BR, XR) are all higher than those of the corresponding purple fruits (DP, BP, XP), further confirming that red fruits exhibit superior comprehensive nutritional quality.

Al, Val, Ca, V, and Cr were identified as the signature components for evaluating the fruit quality of *Nitraria* plants. These five indicators were taken as the core factors for quality evaluation and substituted into the average membership function formula: $\mu =\left(1/n\right)\sum_{i}^{n}=1\mu_{i}$ to calculate the average membership function value of fruits from three *Nitraria* species. As shown in Figure 8, the ranking of comprehensive nutritional quality scores was as follows: XP (0.937) > XR (0.398) > DR (0.255) > DP (0.238) > BR (0.208) > BP (0.146). The comprehensive score of purple fruits of *N. sibirica* (XP) was significantly higher than that of other samples, and its membership index was closer to 1.0, indicating that it had the optimal nutritional quality. This result is consistent with the conclusion proposed by Zhang et al. that the membership function method can effectively realize the comprehensive evaluation of crop nutritional quality, and the closer the membership index is to 1, the better the quality. It also mutually confirms with the results of significance analysis, cluster analysis, and PCA mentioned earlier, verifying the reliability of the evaluation results [41].

Further analysis showed that red and purple fruits of *N. sibirica* exhibited significant advantages in the contents of positive nutritional indicators (Al, Val, Ca, V). *N. roborowskii* had a decreased comprehensive quality score due to the enrichment of toxic element Cr, but it did not exceed the limit of 0.5 mg/kg specified in the National Food Safety Standard of China (GB2762‐2022). *N. tangutorum* was inferior to the other two species due to insufficient Ca accumulation. This quality distribution pattern clearly reveals the quality differences among different species of the genus *Nitraria* and is consistent with the research conclusions proposed by Rolić et al. and Giedyk and Gryko that heavy metal elements such as Cr reduce food quality and Ca is a core nutritional indicator of berries [36, 39]. The average membership values of red fruits (XR) and purple fruits (XP) of *N. sibirica* were significantly higher than those of other groups, indicating that they had the optimal comprehensive nutritional quality.

Within the same species, the average membership values of red fruits (DR, BR, XR) were higher than those of their corresponding purple fruits (DP, BP, XP), further confirming that red fruits have better comprehensive nutritional quality. The evaluation results of the membership function method clarified that Cr and Ca are the key negative factors restricting the improvement of fruit quality of *N. roborowskii* and *N. tangutorum*, respectively, providing a direction for the quality research of *Nitraria* plants and expanding the application of this method in the quality evaluation of desert berries.

## 3. Conclusions

This study systematically analyzed the amino acid and mineral element compositions and nutritional quality of fruits of different colors from three *Nitraria* species in Qinghai Province, and compared and verified the results with existing studies on desert plants and berry nutrition. The main results are as follows.

The contents of TAAs, EAAs, NEAAs, and total mineral elements in *N. sibirica* were significantly higher than those in *N. roborowskii* and *N. tangutorum*. The proportion of EAAs in its purple fruits was close to the FAO ideal protein standard, and it was rich in mineral elements such as Zn and Fe, showing prominent nutritional advantages, confirming that *N. sibirica* is a superior germplasm.

The effect of fruit color on nutrient accumulation was species‐specific: Red fruits had higher contents of NEAAs and TAAs, while the effect of color on EAAs varied with species, and mineral elements were not significantly affected by color. This result is consistent with the anthocyanin metabolic competition mechanism and makes up for the deficiency in the research on the color effect of *Nitraria*.

Chemometric analysis showed that there was a significant synergistic accumulation between amino acids and mineral elements in *Nitraria* fruits, species was the main factor affecting nutritional quality, and five core signature components (Al, Val, Ca, V, Cr) were screened out, improving the quality evaluation system.

Comprehensive evaluation indicated that purple fruits of *N. sibirica* had the optimal nutritional quality and were high‐quality germplasm resources for the development of characteristic functional foods.

This study supplements the nutritional data of *Nitraria* plants in alpine desert areas, improves their nutrient accumulation rules and quality evaluation system, provides theoretical and data support for the development of *Nitraria* resources, breeding of fine varieties, and industrial development, and also provides a reference for similar berry research.

## Author Contributions

Changmao Chen: sample collection, experimental operation, methodology, data analysis, and manuscript writing and revision. Yu Tang: sample collection, methodology, and data analysis. Yang Yang: survey and data collation. Xiuhua Guo: experimental operation and data arrangement. Yubi Zhou: sample collection, experimental design, manuscript writing guidance and overall content review, project management, and funding acquisition. Jie Wang: sample collection, experimental guidance, project management, and funding acquisition.

## Funding

This study received funding from Youth Project of Basic Research Plan of Qinghai Province (2024‐ZJ‐944).

## Disclosure

All authors have read and agreed with the content of the paper, confirming its accuracy and completeness.

## Conflicts of Interest

The authors declare no conflicts of interest.

## Supporting Information

The supporting information for this article includes the following files, which provide detailed experimental methods and additional data to support the results of this study.

Materials and Methods.docx: Detailed information on sample collection and preparation, instruments and reagents, determination methods for amino acids, mineral elements, and total phenols and anthocyanins, as well as data analysis procedures.

Tables S1–S5: Determination results of total phenols and anthocyanins (S1), method validation parameters for amino acids (S2), recovery and precision of volatile amino acids (S3), recovery rates of ICP‐MS internal standards (S4), and method validation parameters for mineral elements and anions (S5).

Amino Acid Quality Control Data.xlsx (Table S6): Quality control data for amino acid determination, including intra‐assay precision (RSD) of 17 amino acids.

Coefficients of variation of amino acids and mineral elements.xlsx (Tables S7, S8): CV analysis of amino acid and mineral element contents in all *Nitraria* fruit samples, used to evaluate experimental repeatability.

## Supporting information


**Supporting Information** Additional supporting information can be found online in the Supporting Information section.

## Data Availability

The data that support the findings of this study are available from the corresponding author upon reasonable request.
