# Supplementary material for: Amino Acid and Mineral Element Analysis and Quality Evaluation of Different Color Fruits of Nitraria in Qinghai
Source: Int J Food Sci. 2026 Apr 6;2026:7405684. doi: 10.1155/ijfo/7405684 (PMC13053940; doi:10.1155/ijfo/7405684)
Supplement: Supplementary file 1 — Supporting Information Additional supporting information can be found online in the Supporting Information section. [file IJFO-2026-7405684-s001.zip › Materials and Methods.docx]

1. Materials and Methods

1.1 Sample Collection and Preparation

Fruits of three *Nitraria* species with different colors were used as experimental materials, which were identified by Professor as *Nitraria* roborowskii Kom. (D), *Nitraria tangutorum* Bobrov. (B), and *Nitraria sibirica* Pall. (X). The samples were designated as follows: *N. sibirica* red fruit (XR), *N. sibirica* purple fruit (XP), *N. tangutorum* red fruit (BR), *N. tangutorum* purple fruit (BP), *N.* roborowskii red fruit (DR), and *N.* roborowskii purple fruit (DP).

Each *Nitraria* species was subjected to three biological replicates for detection. The average value of the three biological replicate data was used as the data analysis measurement value for each sample. The experimental materials were collected during the *Nitraria* harvesting season in May 2024 from the saline-alkali land near Xiaochaidan Lake, Xitieshan Town, Golmud City, Haixi Tibetan Autonomous Prefecture, Qinghai Province, China (37.46°N, 95.61°E, altitude 2830 m). All tested species grew under the same environmental conditions, with an annual precipitation of 10.16 mm, an annual average temperature of 5℃, and an effective accumulated temperature of 68℃.

To ensure the consistent physiological maturity of the fruit samples, their maturity was determined based on appearance color and fruit status(Kong et al., 2024): red fruit samples were required to have a fully red and bright fruit surface; purple fruit samples were required to have a fully purple or purple-black and uniform fruit surface. All collected fruits were in a state of full maturity, plump and juicy, without rot, deterioration, or mechanical damage (Figure 1). During collection, healthy fruits with uniform size, no diseases or insect pests, no bird pecking, and no mechanical damage were selected. They were carefully cut at the pedicel with clean scissors to avoid squeezing the fruits, and fruits on the branches were randomly collected from the same plant.

On the day of harvest, the samples were transported back to the laboratory in the dark, immediately gently rinsed with ultrapure water to remove surface dust and impurities, and the surface moisture was blotted dry with filter paper. The fresh fruits were quickly stored in an ultra-low temperature freezer at -40℃, then dried at 40℃ to a constant weight. The dried materials were crushed into powder, passed through a 40-mesh sieve, placed in a desiccator, and stored at room temperature in the dark for later use.

1.2 Instruments and Reagents

Instruments:Optima 7000 DV inductively coupled plasma optical emission spectrometer (Perkin Elmer, USA)MAR S6 microwave digestion system (CEM, USA)VB20 acid digestion device (Lab Tech, USA)UPT series ultrapure water system (Chengdu Ultrapure Technology Co., Ltd.)PL 203 electronic analytical balance (Mettler-Toledo Instruments Co., Ltd.)Biochrom 30+ amino acid analyzer (DKSH Business (China) Co., Ltd.)KQ-100E ultrasonic cleaner (Kunshan Ultrasonic Instruments Co., Ltd.)Molement ultrapure water system (Shanghai Molle Biotechnology Co., Ltd.)DKB-501A super constant temperature water bath (Shanghai Jinghong Experimental Equipment Co., Ltd.)ICP 5000 inductively coupled plasma optical emission spectrometer (Focused Photonics (Hangzhou) Inc.)UV-2600 UV-Vis spectrophotometer (Shimadzu, Japan)TDL-5-A centrifuge (Shanghai Anting Scientific Instrument Factory)

1.3 Reagents

Petunidin-3-O-(6-p-coumaroyl)rutinoside-5-O-glucoside standard (purity≥98%, Shanghai Yuanye Bio-Technology Co., Ltd., batch No.: Y20250312);Gallic acid standard (purity≥99%, Sigma-Aldrich, batch No.: G7384);Folin-Ciocalteu reagent (Sinopharm Chemical Reagent Co., Ltd., batch No.: 20250408);Potassium chloride, sodium acetate, anhydrous ethanol, etc., were of analytical grade;Na₂CO₃ (analytical grade); NaHCO₃ (analytical grade).

Glucose reference substance (National Institute for the Control of Pharmaceutical and Biological Products, China);A mixed standard solution of 17 amino acids at a concentration of 2.5 μmol/mL: aspartic acid (Asp), glutamic acid (Glu), serine (Ser), histidine (His), glycine (Gly), threonine (Thr), arginine (Arg), alanine (Ala), tyrosine (Tyr), cystine (Cys), valine (Val), methionine (Met), phenylalanine (Phe), isoleucine (Ile), leucine (Leu), lysine (Lys), proline (Pro) (DKSH Business (China) Co., Ltd.).

Single-element standard solutions of K, Na, Ca, Mg, S, and P were prepared at a concentration of 10 mg/mL. Single-element standard solutions of V, Cr, Cd, Co, Cu, Fe, Mn, Ti, Sr, Li, B, and Al were prepared at a concentration of 1000 μg/mL. An 18-element mixed standard solution of rare earth elements (including Y, La, Ce, and Nd) at 1000 μg/mL was purchased from the National Analysis Center for Iron and Steel (NACIS).

1.4 Determination Methods

1.4.1 Determination of Anthocyanins

The anthocyanin content was determined using the AOAC official method 2005.02 (pH differential method) ^[45]^.1.0 g of dried sample was weighed and extracted with 20 mL of methanol containing 0.1% HCl in the dark for 24 h. After filtration, the filtrate was made up to 25 mL.1 mL of the extract was mixed with 9 mL of 0.025 M KCl buffer (pH 1.0) and 9 mL of 0.4 M sodium acetate buffer (pH 4.5), respectively, and incubated in the dark at room temperature for 15 min.Using the corresponding buffer as a blank, absorbance was measured at 520 nm and 700 nm. The corrected absorbance difference was calculated as:

$$\text{X=}\frac{\text{A×MW×}\text{V}_{\text{1}}\text{×V}}{\text{m×ε×l×10}}$$

X: Content of petunidin-3-O-(6-p-coumaroyl)rutinoside-5-O-glucoside in the sample (mg/g)

A: Corrected absorbance difference

MW: Molar mass of petunidin-3-O-(6-p-coumaroyl)rutinoside-5-O-glucoside, 934.4 g/mol

V1: Volume made up during colorimetry (mL)

V: Total volume of the extract (mL)

ε:Molar extinction coefficient ofpetunidin-3-O-(6-p-coumaroyl)rutinoside-5-O-glucoside, 26900 L/(mol·cm)

I: Path length of the cuvette (cm)

m: Mass of the sample (g)

Anthocyanin content was expressed as petunidin-3-O-(6-p-coumaroyl)rutinoside-5-O-glucoside equivalents (mg GAE/kg DW), and the results are shown in Table S1.

1.4.2 Determination of Total Phenols

The total polyphenol content was determined using the Folin-Ciocalteu spectrophotometric method(Lee, Xie, Duan, Ng, & Suleria, 2024):0.5 g of dried sample was extracted with 10 mL of 70% ethanol under ultrasonic conditions (40 ℃, 200 W) for 30 min, then centrifuged at 8000 r/min for 15 min. The supernatant was collected, and the extraction was repeated twice. The combined filtrates were made up to 25 mL.0.5 mL of the extract was mixed with 2.5 mL of 10% Folin-Ciocalteu reagent, and allowed to stand for 6 min. Then, 2.0 mL of 7.5% sodium carbonate solution was added, and the mixture was made up to 10 mL with distilled water. After incubation in the dark at room temperature for 90 min, absorbance was measured at 760 nm.Using gallic acid as the standard, a calibration curve was constructed. The total polyphenol content was expressed as gallic acid equivalents (mg GAE/kg DW), and the results are shown in Table S1.The content was calculated using the following formula:

$$\text{X=}\frac{\text{C×}\text{V}_{\text{1}}\text{×}\text{V}_{\text{3}}}{\text{m×}\text{V}_{\text{2}}}\text{×f×100}$$

X: Total polyphenol content in the sample

C: Concentration of the test solution obtained from the calibration curve (mg/mL)

V1: Volume of the sample solution made up (mL)

V2: Volume of sample solution taken for colorimetric determination (mL)

V3: Volume made up during colorimetric determination (mL)

m: Mass of the sample weighed (g)

f: Dilution factor

Table S1. Analysis of total polyphenols and anthocyanins in different *Nitraria* species

| Sample | Totalpolyphenols（g/100） | Anthocyanins（g/100） |
| --- | --- | --- |
| DR | 0.028±0.001af | 0.021±0.002a |
| DP | 0.033±0.002b | 0.078±0.003b |
| BR | 0.051±0.001c | 0.074±0.001c |
| BP | 0.027±0.001d | 0.014±0.002de |
| XR | 0.043±0.001e | 0.012±0.002de |
| XP | 0.079±0.003af | 0.118±0.001f |

Note: Table S1. Contents of total polyphenols and anthocyanins in different *Nitraria* samples. Data are presented as mean±standard deviation(n=3). Different lowercase letters within the same column indicate significant differences among samples (P<0.05). DR : *N roborowskii* red fruit, DP : *N roborowskii* purple fruit, BR : *N tangutorum* red fruit, BP : *N tangutorum* purple fruit, XR : *N sibirica* red fruit, XP : *N sibirica* purple fruit. Two key trends are observed:

1.The XP sample exhibited the highest contents of both total polyphenols and anthocyanins, which were significantly higher than those of most other samples.

2.A positive correlation trend was observed between total polyphenols and anthocyanins across different samples, with samples such as XP, BR, and DP showing relatively high levels of both components, while BP and XR had lower contents.

1.4.3 Determination of Amino Acid Content

The determination was performed according to the method described in GB 5009.124-2016 National Standard for Food Safety: Determination of Amino Acids in Foods(Li et al., 2025).

Accurately weigh 0.500 g of the pulverized sample into a hydrolysis tube. Add 5.0 mL of hydrochloric acid solution, then make up to approximately 10.0 mL with 6 mol/L hydrochloric acid solution, followed by the addition of 2 drops of phenol. Place the hydrolysis tube in a refrigerant for 5 min, connect it to a vacuum pump, evacuate to near 0 Pa, and then fill with nitrogen. Repeat the evacuation-nitrogen filling process three times, then seal the tube or tighten the screw cap under a nitrogen atmosphere.

Place the sealed hydrolysis tube in a thermostatic electric blast oven at 103 ℃ for hydrolysis for 22 h. Remove the tube, allow it to cool to room temperature, and open it. Filter the hydrolysate into a 50 mL volumetric flask, rinse the hydrolysis tube with pure water, and combine the rinsings with the hydrolysate. Dilute to the mark with pure water and mix thoroughly by shaking.

Accurately transfer 1.0 mL of the filtrate to a 15.0 mL test tube, and evaporate to dryness under reduced pressure at 40 ℃ using a tube concentrator. Dissolve the residue with 1.0 mL of pure water, continue to evaporate under reduced pressure until completely dry. Add 1.0 mL of sodium citrate buffer solution (pH 2.2) to the dried tube to dissolve the residue, mix thoroughly by shaking, filter the solution through a 0.22 μm membrane filter, and transfer it to an instrument sample vial to obtain the sample solution for instrumental analysis.

Instrumental conditions:

Column: Sulfonic acid-type cation exchange resin column (15 cm in length)

Detection wavelengths: 570 nm and 440 nm

Mobile phase: Citric acid-sodium citrate buffer, flow rate 0.25 mL/min

Column temperature: 53 ℃, column pressure: 9.7–10.1 Pa

Ninhydrin solution flow rate: 0.3 mL/min, pump pressure: 0.5 bar

Reaction bath temperature: 98 ℃

Injection volume: 20 μL

Analysis time: 45 min

Two parallel and accurately weighed aliquots of *Nitraria* fruit were hydrolyzed and derivatized for the parallel determination of 17 amino acids. Using the peak area as the ordinate and the concentration of each amino acid solution as the abscissa, the validation experimental data for each amino acid were obtained, as shown in Table S2.

Table S2. Method validation parameters for amino acid analysis in Nitraria samples

| Amino Acid | Linear Regression Equation | Correlation Coefficient (R²) | Linear Range (μmol/L) | RSD (%) | Limit of Detection (g/100 g) | Limit of Quantitation (g/100 g) |
| --- | --- | --- | --- | --- | --- | --- |
| Asp | y=43.82x+2.15 | 0.9972 | 2.5–100 | 4.2 | 0.00013 | 0.00036 |
| Thr | y=34.65x+1.02 | 0.9968 | 2.5–100 | 5.1 | 0.00014 | 0.00048 |
| Ser | y=42.28x+1.87 | 0.9959 | 2.5–100 | 4.8 | 0.00018 | 0.0006 |
| Glu | y=37.44x+1.53 | 0.9965 | 2.5–100 | 3.7 | 0.00024 | 0.0007 |
| Gly | y=67.30x+3.21 | 0.9947 | 2.5–100 | 4.5 | 0.00025 | 0.00084 |
| Ala | y=48.10x+2.45 | 0.9983 | 2.5–100 | 2.9 | 0.0029 | 0.0097 |
| Cys | y=5.78x+0.32 | 0.9935 | 2.5–100 | 5.8 | 0.0001 | 0.0002 |
| Val | y=41.01x+1.95 | 0.9961 | 2.5–100 | 4.1 | 0.00012 | 0.00032 |
| Met | y=41.32x+2.01 | 0.9942 | 2.5–100 | 5.4 | 0.0023 | 0.0075 |
| Ile | y=37.79x+1.68 | 0.9955 | 2.5–100 | 4.6 | 0.00043 | 0.0013 |
| Leu | y=28.22x+1.34 | 0.9976 | 2.5–100 | 3.5 | 0.0011 | 0.0036 |
| Tyr | y=35.21x+1.72 | 0.9963 | 2.5–100 | 4.3 | 0.0028 | 0.0095 |
| Phe | y=38.78x+1.89 | 0.9957 | 2.5–100 | 4 | 0.0025 | 0.0083 |
| Lys | y=49.46x+2.51 | 0.9974 | 2.5–100 | 4.7 | 0.00013 | 0.00044 |
| His | y=46.78x+2.33 | 0.9949 | 2.5–100 | 5.3 | 0.00059 | 0.002 |
| Arg | y=38.84x+1.92 | 0.9967 | 2.5–100 | 4.4 | 0.002 | 0.0065 |
| Pro | y=20.23x+0.98 | 0.9981 | 2.5–100 | 3.2 | 0.0026 | 0.0087 |

Note: Table S2. Method validation parameters for amino acid analysis in *Nitraria* samples, including linear regression equations, correlation coefficients, linear ranges, relative standard deviations (RSD), limits of detection (LOD), and limits of quantitation (LOQ). Two key observations are noted:

1.All amino acids showed excellent linearity (R²>0.993) within the range of 2.5–100 μmol/L, indicating the method is suitable for quantitative analysis.

2.The method demonstrated high sensitivity, with LOD values ranging from 0.0001 to 0.0026 g/100·g and LOQ values from 0.0002 to 0.0097 g/100·g, enabling accurate detection of trace amino acids.

Amino acid hydrolysis and correction for volatile loss: After freeze-drying, approximately 20 mg of sample was accurately weighed and hydrolyzed with 6 mol/L HCl containing 0.1% phenol at 110 °C for 24 h under nitrogen. After hydrolysis, the solution was filtered through a 0.22 μm membrane filter, and the filtrate was analyzed using an automatic amino acid analyzer. To correct for the loss of volatile amino acids during hydrolysis, a known amount of L-norleucine was added as an internal standard before hydrolysis. Correction factors were calculated from the internal standard recovery rates to adjust the target amino acid contents. The recoveries of all volatile amino acids were within 85%–115%, and the RSD of replicate experiments was less than 5%, ensuring the accuracy of the results (see Table S3 for details).

Table S3. Recovery and precision validation of volatile amino acids in *Nitraria* samples

| Amino acid | Theoretical spiked amount (µg) | Measured average content (µg) | Recovery (%) | RSD |
| --- | --- | --- | --- | --- |
| Cys | 10 | 9.2 | 92 | 3.2 |
| Met | 10 | 9.5 | 95 | 2.8 |
| Thr | 10 | 9.8 | 98 | 2.1 |
| Ser | 10 | 9.7 | 97 | 2.5 |

Note:Table S3. Recovery and precision of volatile amino acids (Cys, Met, Thr, Ser) in *Nitraria* samples. All recoveries were within the acceptable range of 85%–115%, and the relative standard deviations (RSD) of replicate experiments were less than 5%, confirming the accuracy and reliability of the method for correcting losses of volatile amino acids during acid hydrolysis.

1.4.4 Determination of Mineral Element Content

The mineral element content was determined using the wet digestion method specified in GB 5009.268-2016 National Standard for Food Safety: Determination of Multiple Elements in Foods [41].

Accurately weigh 3 mg of the pulverized sample into a 100 mL conical flask. Add 20 mL of a nitric acid:perchloric acid (9:1) mixed acid and soak overnight. Heat the flask on a hot plate at 250 ℃ for digestion. If carbonization occurs during digestion, add an additional 1 mL of nitric acid. After complete digestion, evaporate the solution until it becomes colorless or pale yellow. After cooling, transfer the digest to a 25 mL volumetric flask using grade 1 water, dilute to the mark, and mix thoroughly for analysis. A reagent blank was prepared in parallel.

Standard curve preparation:From the 1000 μg/mL standard stock solutions of each element, transfer 0.10 mL into a 100.0 mL volumetric flask and dilute to the mark with 1% nitric acid to prepare a 1 μg/mL working solution. The internal standards were In, Ge, Bi, and Sc, each at a concentration of 500 μg/L. For details, see Table S4.

Instrumental conditions for ICP:

RF power: 1150 W, Nebulizer gas flow rate: 0.6 L/min, Auxiliary gas flow rate: 3 L/min, Cooling gas flow rate: 2 L/min, Flush pump speed: 100 r/min, Analysis pump speed: 50 r/min, Flush time: 15 s, Viewing mode: axial, Number of repeated measurements: 3

Instrumental conditions for ICP-MS:RF power: 1500 W, Nebulizer gas flow rate: 1.01 L/min, Auxiliary gas flow rate: 0.9 L/min, Number of scans: 3, Stabilization time: 40 s, Number of repeated measurements: 3, Sample uptake time: 30 s, Spray chamber temperature: 2 ℃.

Table S4. Recovery rates of internal standards (Sc, Ge, In, Bi) in *Nitraria* samples

| Recovery rate % | Sc | Ge | In | Bi |
| --- | --- | --- | --- | --- |
| DR | 104.5 | 105.5 | 102.4 | 103.2 |
| DR | 101.8 | 104.8 | 101.7 | 103.5 |
| DR | 104.9 | 106.3 | 103.1 | 103.2 |
| DP | 105.9 | 108.8 | 104.8 | 103.6 |
| DP | 97.6 | 102.3 | 98.8 | 98.7 |
| DP | 101.8 | 105.1 | 101.3 | 101.5 |
| BR | 114.4 | 96.5 | 99.9 | 113.0 |
| BR | 105.4 | 109.0 | 104.8 | 102.6 |
| BR | 103.9 | 99 | 96 | 96.6 |
| BP | 103.7 | 107.7 | 104.1 | 105.1 |
| BP | 105.0 | 108.5 | 105.7 | 106 |
| BP | 104.4 | 108.1 | 104.1 | 105.9 |
| XR | 102.8 | 105.6 | 101.4 | 100.1 |
| XR | 104.1 | 107.1 | 102.1 | 101.1 |
| XR | 105.3 | 105.9 | 101.6 | 100.8 |
| XP | 104.3 | 106.8 | 102.2 | 101.3 |
| XP | 107.3 | 108.0 | 103.1 | 101.1 |
| XP | 108.1 | 108.3 | 103.4 | 102.6 |

Note:Table S4. Recovery rates of internal standards (Sc, Ge, In, Bi) in different *Nitraria* samples. All recoveries were within the acceptable range of 85%–115%, demonstrating the reliability and accuracy of the ICP-MS method for mineral element quantification in *Nitraria* samples.

Target anions in water samples were determined by ion chromatography ^[47].^The instrumental conditions were as follows: anion exchange column SH-AC-3; conductivity detector; flow rate 1.000 mL/min; injection volume 25.0 μL; column temperature 25 ℃; detection cell temperature 35 ℃; suppressor current 50 mA.The eluent was prepared by dissolving 0.212 g Na₂CO₃ and 0.672 g NaHCO₃ in 1 L of ultrapure water, filtered through a 0.22 μm membrane, degassed, and used freshly.

For sample pretreatment, water samples were filtered through a 0.22 μm membrane to remove suspended particles.Water samples with high hardness were pretreated with purification cartridges to remove interfering ions such as Ca²⁺ and Mg²⁺.Samples containing organic compounds were passed through a C18 cartridge to remove hydrophobic organic substances, followed by filtration through a 0.22 μm membrane.The filtrate was stored at 4℃ in the dark and analyzed within 24 h.

For calibration, a series of standard solutions with gradient concentrations of target anions were prepared and analyzed under the above conditions.Linear regression equations were established with peak area as the ordinate and ion concentration as the abscissa, ensuring a correlation coefficient R²≥0.999.

After the instrument baseline was stabilized, 10 mL of pretreated water sample was placed in the autosampler and analyzed according to the programmed method.Each sample was determined in triplicate.The peak areas were recorded and substituted into the calibration curve to calculate the ion concentrations, and the average values were reported.Details are shown in Table S5.

Table S5. Method validation parameters for elemental and anionic analysis in *Nitraria* samples

| Element | Linear Regression Equation | Correlation Coefficient (R²) | Limit of Detection (ug/g) | Limit of Quantitation (ug/g) |
| --- | --- | --- | --- | --- |
| Ca | y=1.23210E6x+4.03288E5 | 0.99991 | 0.3000 | 3.0000 |
| K | y=1.87391E6x-9.23207E5 | 0.99949 | 0.3000 | 3.0000 |
| P | y=3.043E1x+3.711E2 | 0.9994 | 0.1926 | 1.9260 |
| S | y=1.357E1x+1.193E4 | 1 | 5.4230 | 54.2300 |
| Mg | y=1.43333E5x-7572.51700 | 0.99998 | 0.1000 | 1.0000 |
| Na | y=3.04628E6x-1.36341E6 | 0.99959 | 0.3000 | 3.0000 |
| Cl⁻ | y=0.9976x+0.02 | 0.9999 | 0.3000 | 3.0000 |
| V | y=9.341E3x+8.878E2 | 0.9999 | 0.0005 | 0.0055 |
| Cr | y=6.082E-2x+9.070E-3 | 0.9999 | 0.0124 | 0.1235 |
| Co | y=1.536E-1x+2.596E-4 | 1 | 0.0003 | 0.0031 |
| Cu | y=9.370E-2x+1.080E-1 | 1 | 0.0110 | 0.1095 |
| Fe | y=5.902E-2x+4.802E-1 | 0.9999 | 0.0200 | 0.2000 |
| Mn | y=4.449E-2x+2.073E-2 | 1 | 0.0059 | 0.0592 |
| F⁻ | y=1.0040x-0.0292 | 0.9999 | 0.0000 | 0.0000 |
| Ti | y=5.161E4x+2.444E1 | 0.9999 | 0.0001 | 0.0006 |
| Sr | y=9.833E-2x+7.527E-3 | 1 | 0.0021 | 0.0210 |
| Li | y=3.257E2x+8.945E2 | 0.9998 | 0.0710 | 0.7098 |
| B | y=1.064E2x+4.233E3 | 1 | 0.3395 | 3.3950 |
| Al | y=2812E-3x+1.134E-2 | 0.9999 | 0.0951 | 0.9512 |
| Nd | y=1.224E3x+2.444E1 | 1 | 0.0031 | 0.0309 |
| Y | y=2.119E-2x+5.693E-4 | 1 | 0.0004 | 0.0043 |
| La | y=5.626E3x+4.534E2 | 0.9999 | 0.0021 | 0.0208 |
| Ce | y=5.940E3x+2.279E4 | 1 | 0.1068 | 1.0680 |
| NO₃⁻ | y=0.9976x+0.02 | 0.9999 | 0.3000 | 3.0000 |
| SO₄²⁻ | y=0.9976x+0.02 | 0.9999 | 0.3000 | 3.0000 |
| PO₄³⁻ | y=0.9976x+0.02 | 0.9999 | 0.3000 | 3.0000 |
| Cd | y=2.855E-3x+6.130E-5 | 0.9999 | 0.0008 | 0.0080 |

Note :Table S5. Method validation parameters for elemental and anionic analysis in *Nitraria* samples, including linear regression equations, correlation coefficients, limits of detection (LOD), and limits of quantitation (LOQ). All analytes exhibited excellent linearity (R² ≥ 0.9994) within the tested concentration range, and the method demonstrated high sensitivity with low LOD and LOQ values, confirming its suitability for the accurate quantification of elements and anions in Nitraria samples.

1.5 Data Analysis

Data analysis and graphing were performed using IBM SPSS Statistics 26.0 and Origin 2021 software. All experimental data were expressed as the mean ± standard deviation (x̄±SD) with three biological replicates (n = 3). Significant differences among groups were determined by one-way analysis of variance (ANOVA) followed by the least significant difference (LSD) multiple comparison test. Differences were considered significant at ***P*<0.05** and highly significant at ***P*< 0.01**. Prior to multivariate analysis, data were standardized using the Z-score method.

Kong, F., Li, X., Zhao, J., Zhou, Z., Zhou, C., Yan, Z., Wei, Y. (2024). Molecular phylogenetic and biogeographical analysis of Nitraria based on nuclear and chloroplast DNA sequences. *Plant Systematics and Evolution, 45*(01), 63-71. doi:10.13386/j.issn1002-0306.2023020064

Lee, Z. J., Xie, C., Duan, X., Ng, K., & Suleria, H. A. R. (2024). Optimization of Ultrasonic Extraction Parameters for the Recovery of Phenolic Compounds in Brown Seaweed: Comparison with Conventional Techniques. *Antioxidants, 13*(4). doi:10.3390/antiox13040409

Li, Y., Wang, S., Zhao, Q., Wang, Q., Yang, Z., & Jia, Q. (2025). Integrating metabolomics and transcriptomics comprehensively reveals the global metabolic differences in three species of Nitraria berries. *Sci Rep, 15*(1), 15507. doi:10.1038/s41598-025-00445-0
